# Association of sarcopenia and observed physical performance with attainment of multidisciplinary team planned treatment in non-small cell lung cancer: an observational study protocol

**DOI:** 10.1186/s12885-015-1565-6

**Published:** 2015-07-24

**Authors:** Jemima T. Collins, Simon Noble, John Chester, Helen E. Davies, William D. Evans, Jason Lester, Diane Parry, Rebecca J. Pettit, Anthony Byrne

**Affiliations:** 1 Department of Palliative Medicine, University Hospital Llandough, Penarth, UK; 2 Cardiff University, Cardiff, UK; 3 Velindre Cancer Centre, Cardiff, UK; 4 Department of Respiratory Medicine, University Hospital Llandough, Penarth, UK; 5 Department of Medical Physics and Clinical Engineering, University Hospital of Wales, Cardiff, UK

**Keywords:** Sarcopenia, Muscle mass, Physical performance, Lung cancer, Fitness

## Abstract

**Background:**

Non-small cell lung cancer (NSCLC) frequently presents in advanced stages. A significant proportion of those with reportedly good ECOG performance status (PS) fail to receive planned multidisciplinary team (MDT) treatment, often for functional reasons, but an objective decline in physical performance is not well described. Sarcopenia, or loss of muscle mass, is an integral part of cancer cachexia. However, changes in both muscle mass and physical performance may predate clinically overt cachexia, and may be present even with normal body mass index. Physical fitness for treatment is currently subjectively assessed by means of the PS score, which may be inadequate in predicting tolerance to treatment. This study aims to evaluate whether measuring physical performance and muscle mass at baseline in NSCLC patients, in addition to PS score, is able to predict commencement and successful completion of MDT-planned treatment.

**Methods/design:**

This is a prospective, single-centre exploratory study of NSCLC patients attending a Rapid Access Lung Cancer clinic. Baseline data collected are (methods in brackets): physical performance (Short Physical Performance Battery), muscle mass (bioelectrical impedance ± dual energy x-ray absorptiometry), patient and physician-assessed PS (ECOG and Karnofsky), nutritional status and presence of cachexia. Longitudinal data consists of receipt and completion of MDT treatment plan. The primary outcome measure is commencement of MDT-planned treatment, and important secondary outcomes include successful completion of treatment, length of stay in surgical patients, and risk of chemotherapy- and radiotherapy-related side effects.

**Discussion:**

A more comprehensive assessment of phenotype, particularly with regards to physical performance and muscle mass, will provide additional discriminatory information of patients’ fitness for treatment. If positive, this study has the potential to identify targets for early intervention in those who are at risk of deterioration. This will subsequently enable optimisation of performance of patients with NSCLC, in anticipation of systemic treatment.

## Background

Lung cancer is a common disease, with a global incidence of 1.61 million in 2008 [1, 2]. Non-small cell lung cancer (NSCLC) is the most common cell type, with the majority of patients presenting with advanced disease. Despite advances in anticancer treatments, the mortality risk of lung cancer remains high worldwide, with an annual mortality to incidence ratio of 88 % [3]. In the United Kingdom, annual mortality from NSCLC is similarly poor, with an overall 5-year survival rate of 8.6 % [4].

Despite this, survival rates in NSCLC are consistently superior in patients who receive specific anticancer treatment, compared to those who do not [5–7]. Potentially curative treatment strategies using surgery, radical radiotherapy or multimodality treatment is feasible in a minority of patients (12–19 %), whilst systemic anti-cancer therapy is recommended for those with incurable disease [8–10]. Multidisciplinary team (MDT) assessment of fitness for optimal treatment – be it surgery, radiotherapy or chemotherapy, singly or in combination – is crucial, as all these modalities are poorly tolerated by patients with low baseline physical function [11, 12]. Currently assessment of fitness for treatment is based on initial clinical examination, consideration of co-morbidities and clinician assessment of functioning, most often reported as Eastern Cooperative Oncology Group (ECOG) or Karnofsky performance status (PS) [13, 14]. Evidence suggests that PS assessment at baseline is predictive of overall survival [15, 16]. However, there is less evidence of sensitivity to change over time or of reliable correlations with tolerance of treatment. Furthermore, PS assessment is subjective, with reports of inter-observer variability [17, 18], and there may only be a modest correlation between PS and observed physical performance [19]. Most tellingly, recent evidence also suggests that up to 33 % of NSCLC patients for whom active treatment is recommended by the MDT do not go on to receive it, mainly due to functional reasons [20, 21].

Physical function is largely dependent on maintenance of muscle mass and neuromuscular function. Although weight loss has long been recognised as an adverse feature of cancer cachexia, it is the depletion of skeletal muscle mass – sarcopenia – with or without loss of fat mass, which is now an integral part of the diagnosis of cancer cachexia [22]. Its presence is associated with an increased risk of post-operative morbidity, longer hospital stays, as well as poorer survival [23–26]. Depletion of muscle mass may also give rise to chemotherapy toxicities and a poorer response to chemotherapy. In a study of NSCLC patients, variation in muscle mass per unit of body surface area accounted for up to three times the variation of chemotherapy volume of distribution, thus contributing to under- or over-dosing [26]. Clinically, this may manifest as increased risk of dose-limiting toxic side effects in sarcopenic patients, as has been reported in breast, hepatocellular and renal cancer [27–29].

Whilst these studies attest to the importance of muscle mass, less attention has been paid to muscle function (strength and physical performance) as part of the evaluation of sarcopenia in cancer. Muscle mass is linked to muscle function in a non-linear fashion in the elderly, and consequently muscle mass alone cannot fully account for poor outcomes associated with sarcopenia [30–32]. As a geriatric syndrome, therefore, the importance of overall neuromuscular function is recognised, with the recommendation that sarcopenia be defined as a combination of loss of physical performance as well as muscle mass [33]. However, the value of assessing physical performance in lung cancer, particularly with regards to tolerance of treatment, is not known.

In light of this gap in knowledge, calls have been made for objective assessments of physical performance which have prognostic value, to evaluate tolerance of systemic treatment [34, 35]. The gold standard method of assessing physical fitness (cardiopulmonary exercise testing) is laboratory-based and not always feasible for use in patients with malignancy [36]. In addition, it is not widely available and takes over 2 hours to perform and report one case. Other more commonly used tests such as the 6-minute walk test (6MWT) can be challenging to use in clinical practice and is often reserved as research tools [34, 37]. The term ‘physical performance’ in this context implies the ability to undertake physical tasks which are dependent on different physiological domains [38]. Utilising a measurement tool which assesses overall function is therefore more appropriate, and is in keeping with guidelines for the elderly. The Short Physical Performance Battery (SPPB) is one such test, measuring balance, gait speed, lower limb strength and endurance – all crucial in performing activities of daily living. It is a valid, reliable and feasible measure of physical performance in older people [39, 40]. It is quick to perform, easily replicable, requires little additional equipment, and with basic training can be performed by most healthcare personnel.

In the assessment of sarcopenia, current gold standard techniques of measuring muscle mass include computed tomography (CT) and dual energy x-ray absorptiometry (DXA) [41]. However, these techniques involve radiation and either additional investigations (DXA) or extension of current CT staging scans to the L3 level with additional software and expertise for interpretation. By contrast, bioelectrical impedance (BIA) measures muscle mass quickly and easily in clinic, with no radiation exposure. It operates through the application of a small, painless electrical current through the body, which enables calculation of the proportions of fat and muscle mass [42]. It is one of the key methods for assessment of muscularity cited in the new definition of cancer cachexia [22], and is a practical tool for measurement of body composition in cancer patients [43–45].

Whilst current retrospective evidence suggests that muscle mass depletion is associated with increased treatment-related adverse events, it is unclear to what extent measures of muscle mass as well as function at baseline can predict ability to receive and tolerate treatment, and whether they might outperform current methods of MDT assessment. Understanding this may enable more efficient delivery of treatment to this vulnerable patient group, enabling rationalisation of healthcare provision. In addition, it may identify targets for intervention allowing optimisation of patient performance in anticipation of systemic treatment.

### Study aims and design

#### Primary aim

To explore whether measures of skeletal muscle mass and physical performance, at first presentation, can predict commencement of MDT-planned treatment in patients with NSCLC.

#### Secondary aims

##### In patients with NSCLC

1. To evaluate the value of baseline measurements of muscle mass and physical performance as predictors of successful completion of MDT-planned treatment.
2. To explore the prevalence of low muscle mass (sarcopenia) and its relationship to physical performance and degree of chemotherapy-related side effects.
3. To explore the relationship between PS and muscle mass, physical performance and nutrition status.
4. To evaluate the utility of BIA as a practical clinical tool for estimating muscle mass, using DXA as the gold standard comparator in a sub-group of patients.
5. To assess the feasibility of assessing physical performance using SPPB in the rapid access lung clinic setting.

#### Patient recruitment

A target of 100 participants with new suspected NSCLC are being recruited from the Rapid Access Chest Clinic (RACC) in a single-centre, University Hospital setting. Approximately 410 new patients attend this clinic each year. Of patients attending a RACC, 36–50 % have a subsequent diagnosis of primary lung malignancy, and of this 81 % have NSCLC [46, 47]. These figures suggest that our expected recruitment target of 100 over 18 months is achievable. Introduction to the study will be at the discretion of the chest physicians in the clinic, who will decide when it is appropriate to offer an invitation based on likelihood of NSCLC diagnosis and level of emotional distress.

#### Ethical considerations

It is important to acknowledge some of the ethical challenges this study brings. Patients are recruited before a definitive diagnosis of NSCLC has been made. This emphasises the importance of appropriate participant selection by the chest physicians acting as gatekeepers, who only offer participation in the study based on a high clinical likelihood of NSCLC, patients’ understanding of this and their level of emotional distress. In addition, recruitment on the same day as first presentation can present a challenge in terms of gaining informed consent and timing around other investigations. However, this will enable capture of feasibility and acceptability data, which may be of use to the MDT for planning of future services. Having acknowledged these challenges and ensuring that the Patient Information Leaflet and Consent forms reflect this, the South East Wales Research Ethics Committee granted a favourable opinion in November 2013 (REC reference 13/WA/0254).

#### Sample size

To the best of our knowledge, there are no current studies evaluating the relationship of both muscle mass and physical performance to treatment outcomes for lung cancer patients; therefore power calculations to estimate optimal sample size are not possible.

#### Inclusion criteria

- Patients with non-small cell lung cancer (NSCLC) based on clinical and radiological suspicion.
- Clinician-scored ECOG PS 0–2 at presentation.
- Age 18 years or more.

#### Exclusion criteria

- Presence of implantable cardioverter defibrillator or pacemaker.
- Patients who have had strenuous activity or excessive alcohol within 24 h prior to the study, or patients with significant peripheral oedema, all of which may alter the accuracy of BIA measurements.
- Patients with neurological or physical impediment which would preclude participation in the study.
- Inability to give informed consent due to insufficient ability to understand and communicate in English.

### Study assessments

#### Baseline information

The following information is collected from participants’ medical notes: weight and height, degree of any recent weight loss, co-morbidities including severity of chronic obstructive pulmonary disease (COPD) if present, use of non-steroidal anti-inflammatory medicines (NSAIDs) or steroids, and routine blood test results (haemoglobin, albumin, and C-reactive protein).

#### Muscle mass

Predicted muscle mass (MM) is measured using BIA (Tanita BC-418). Participants are asked to place their hands and feet on to the machine which resembles a weighing scale. A printout of the participant’s body composition is generated, accounting for age and gender. This assessment takes roughly 1 min and is repeated in order to calculate precision. To assess the accuracy of BIA-derived muscle mass, this is compared to whole body DXA (Hologic Discovery A) measurements of lean tissue mass in a subgroup of patients (*n* = 20) (this is performed within 3 days of BIA). The DXA scan involves lying flat in the supine position for 5 min, while the machine’s scanning arm runs over the patient’s body. The estimated radiation of this scan is 4.2 microsieverts, which is comparable to the risk associated with one day’s worth of natural background radiation [48].

#### Physical performance

In order to assess objectively physical performance, the Short Physical Performance Battery (SPPB) is utilised [49]. It consists of balance, gait speed and chair stand tests which take between 7 and 10 min to perform. The balance tests assess the participants’ ability to hold these positions for 10 s: (i) both feet together (side-by-side), (ii) heel of one foot touching the big toe of the other (semi-tandem stand) and (iii) heel of one foot in front of and touching the toes of the other (tandem stand). The gait speed test consists of two timed walks of 4 metres each, at the participant’s usual walking pace. The chair stands test consists of five timed stands from a sitting position without the use of arms. As the SPPB is the most time-consuming portion of the study assessments, we will give participants the option of being tested at home, provided this is done within 3 days. In this instance, the research team will arrange to visit them at a place of their choice e.g. their home.

#### Performance status

Both physician- and patient-assessed PS scores (ECOG and Karnofsky scales) are obtained and noted. For the purposes of patient-assessed PS, lay wording was taken directly from the Cancer Research UK website, with permission [50]. We have omitted the descriptions of 5 on the ECOG scale and 0 on the Karnofsky, to minimise distress.

#### Nutrition status

The Malnutrition Universal Screening Tool (MUST) [51] is a simple and quick method of screening for malnutrition in at-risk patients, based on body mass index, unplanned weight loss and recent nutritional intake. It has been validated in patients with cancer [52].

### Outcome measures

#### Primary outcome measure

The primary outcome measure is defined as a binary outcome – yes or no. This is determined as receipt of the MDT-planned surgical procedure or commencement of MDT-planned radiotherapy or chemotherapy course.

#### Secondary outcome measure

Successful completion of MDT-planned treatment is noted. For chemotherapy this will be defined as receipt of the pre-planned number of treatments at pre-planned dose intensity; for surgery, receipt of the pre-planned intervention and for radiotherapy, completion of the pre-planned number of fractions at the pre-defined dose intensity.

#### Progress through chemotherapy

For the purposes of this study, we will note the type and grade of toxicity by Common Terminology Criteria for Adverse Events (CTCAE) criteria, if any, its frequency and requirement for dose reduction, and the number of cycles of chemotherapy completed.

#### Progress through other treatment courses

We will note the completion of the surgical intervention, length of stay and any post-operative complications. For participants undergoing RT, we will document any radiotherapy toxicities and the number of fractions completed.

### Data analysis

Correlations between independent variables – muscle mass, SPPB scores, age, gender, performance status and treatment receipt and successful completion will be analysed using logistic regression. Any associations between the presence of sarcopenia and the outcome measures of interest will be analysed. For secondary outcomes we will assess the strength of correlations by calculating correlation coefficients and their 95 % confidence intervals. We aim to document the strength of agreement between DXA- and BIA-derived muscle mass values with intraclass correlation coefficient (ICC), where an ICC of more than 0.75 is generally considered excellent.

## Discussion

The majority of patients with lung cancer present in advanced stages, many incurable with a median survival of 9–12 months [53]. For treatment to have its optimum effect, it needs to be initiated in a timely manner, with appropriate patient selection, taking into account levels of physical fitness for treatment. In its latest guidance on lung cancer, the National Institute for Health and Clinical Excellence (NICE) assert that factors predicting success in treatment in patients with borderline fitness levels is as yet unclear. It therefore recommends robust, consistent and meaningful studies into assessments of fitness parameters, with appropriately designed trials [54].

The patient’s journey between first presentation with suspected lung cancer and review by the treating oncologist or surgeon is not well-described; however there is evidence that a significant proportion of patients are unable to receive all of their MDT-planned treatment, for functional reasons [20, 21]. Objective, formalised assessment of physical performance alongside assessment of muscle mass at baseline may provide additional discriminatory information, as an adjunct to PS, which would better inform MDT decisions. There are many candidate tools for assessing physical performance and muscle mass [37, 41]. However, as well as being reliable and having predictive validity, the best methods should also be easily performed in clinical settings, alongside other measurements of body mass index, body surface area and performance status.

As interest intensifies in the role of wider supportive interventions for cancer patients, understanding baseline levels of muscle mass and physical function is of growing importance in defining individualised supportive care plans. There is good evidence that exercise programmes have varied, beneficial outcomes for cancer patients including improved levels of cardio-respiratory fitness, objective performance, self-reported functioning and reduced cancer-related fatigue in systemic treatment [55–57]. Even in NSCLC patients with advanced disease, there is emerging evidence that exercise may have a favourable role [37, 58]. However, it is currently unclear at what time point this should be initiated to obtain greatest benefit, the type and intensity of the intervention and whether early initiation can improve tolerance of treatment in this patient group. A multi-dimensional assessment of physical functioning at baseline would allow early identification of patients at greatest risk of functional decline and thereby stratification for enrolment in exercise programmes or other interventions, in preparation for systemic anticancer treatment.

In summary, measurement of patient performance and muscle mass, using validated and easily replicated tools, may add important objective detail of patients’ physical fitness to the currently utilised PS score and aid MDT decision making. These assessments would enable more accurate prediction of successful attainment of therapeutic goals highlighting patients at potential risk from planned treatments and thereby allowing intervention to be targeted at individual functional domains. This could have potential implications for MDT co-ordination and clinical service provision yet ensure efficient and prudent patient care.
